# To the Editors of the Medical and Physical Journal

**Published:** 1807-06

**Authors:** 


### *To the Editors of the Medical and Physical Journal.*

GENTLEMEN,

HAVING seen repeated instances of the ill effects produced by a promiscuous use of injections for the cure of gonorrhœa, I shall feel obliged by your inserting the following Cases in your next Journal, wherein I have particularized a plan of treatment, of which (before I myself adopted it), I never heard, and from which I have received the greatest satisfaction. If, therefore, by it, I shall save an individual a moment's suffering, my wishes will be crowned with the utmost success.

I am, &c.

W.

*Exeter, May* 10, 1807.

### CASE THE FIRST.

*April* 12, 1807. Mr. A—— B——, a young man æt. 22, of a very irritable habit, subject to frequent attacks of erysipelas, and accustomed to live freely, having contracted a gonorrhœa, applied to me for advice, three weeks after he had received the infection. On examining the penis, the glans and prepuce were much inflamed and very painful, the ardor urinæ almost insupportable; he likewise felt great uneasiness throughout the urethra. I first gave a large dose of the sulphate of magnesia, and desired him to keep the penis constantly wet with the saturnine lotion, to make barley water his constant drink, and avoid every thing inflammatory.

13th. He had passed a restless night, from frequent erections and a violent chordee; ordered a bolus of camphor and opium; continue the lotion.

14th and 15th. Rested better, chordee not so troublesome; repeat the bolus, &c.

17th. The chordee very slight, discharge copious, but more purulent, the glans not so much inflamed, bowels costive; repeat the cathartic lotion and bolus.

20th. The chordee left him; omit the bolus; the ardor urinæ trifling, discharge nearly the same as on the 17th.

24th. Still better; continue the lotion.

30th. Discharge much less, principally in the morning; ardor urinæ left him; ordered an injection of sulphat. zinci gt. iv aq. distillat. ℥ij. to be used every night and morning.

May

*May* 7. Discharge quite disappeared; says, "he is as well as at any time of his life."

### Case the Second.

*April* 16th. Mr. C—— D——, aged 50, a man much addicted to the free use of spirituous liquors, received the infection a fortnight previous to his application to me; he had a chancre on the prepuce, which was much swollen, and had received a partial phymosis the ardor urinæ so great, that, to use his own expression, "it was almost bad enough to take away his life." I ordered him to take salts twice a week, apply the saturnine lotion, take one grain of calomel every night, and desist from every thing inflammatory.

By a constant and steady perseverance in these means, I had the pleasure of pronouncing him perfectly cured on May 8. — C. D. used no injection.

By these means I have cured several others, but it would be tedious to detail their cases.

---

